# Organizational Cronyism as an Antecedent of Ingratiation: Mediating Role of Relational Psychological Contract

**DOI:** 10.3389/fpsyg.2019.01609

**Published:** 2019-07-19

**Authors:** Sadia Shaheen, Muhammad Waseem Bari, Filza Hameed, Muhammad Mudassar Anwar

**Affiliations:** ^1^Lyallpur Business School, Government College University Faisalabad, Faisalabad, Pakistan; ^2^Department of Business Administration, University of Kotli Azad Jammu and Kashmir, Kotli, Pakistan; ^3^Department of Commerce, University of Kotli Azad Jammu and Kashmir, Kotli, Pakistan

**Keywords:** organizational cronyism, ingratiation, relational psychological contract, social exchange theory, public organizations, Pakistan

## Abstract

The present study investigates the relatively less explored construct organizational cronyism as an antecedent of employees’ ingratiation. Moreover, the role of the relational psychological contract as a mediator between organizational cronyism and ingratiation is also examined. The data were collected from employees working in different ministerial offices, such as the ministry of defense production, human rights, parliamentary affairs, petroleum, and natural resources, in Islamabad, Pakistan. Through a convenience sampling approach, 250 employees provided data for this study. Due to sensitivity and less approachability to these organizations, the convenience sampling technique was used. The data were collected in two waves with 12-week intervals. The results confirm that organizational cronyism is significantly related to the relational psychological contract, which in turn results in employees’ ingratiation. The employees who have close ties with their leaders and enjoy extraordinary favors from their leaders, display more compliance behavior as compared to those employees who have distance from their leaders.

## Introduction

Organizational cronyism is known as bestowing of favor to colleagues, friends, and someone who has personal relations with the favor-giver (Turhan, 2014). It is also acknowledged as anti-meritocracy practice and has gained much attention in recent literature. Several scholars have identified numerous antecedents of organizational cronyism, such as particularism, which arises from ingroup bias and results in organizational cronyism (Khatri et al., 2006). Similarly, paternalism gives rise to personal loyalty which also results in organizational cronyism. Ultimately, organizational cronyism results in various attitudinal and behavioral outcomes such as job dissatisfaction, deviant workplace behavior, counterproductive work behavior and low organizational commitment as well as organizational citizenship behavior, and ingratiation (Özsemerci, 2003; Diefenbach, 2009; Asunakutlu and Avci, 2010; Choi, 2011; Karaköse, 2014; Pearce, 2015; Kteily and Bruneau, 2017).

The literature explains two types of organizational cronyism, known as vertical cronyism and horizontal cronyism (Khatri, 2006). Horizontal cronyism refers to the relationships based on favoring others at the same level, designation, and class—e.g., friends, colleagues, classmates, social groups, and unions. On the other hand, vertical cronyism refers to undue favors by a supervisor to a subordinate in an unjustified way and without performance, such as favoring employees by providing better working conditions and undue promotions. The employees who receive undue benefits, favor, and support at the expense of others are known as cronies (Turan, 2015). The employees who are discriminated and do not get benefits from their leader or supervisor because cronies are referred to non-cronies (Turan, 2015). At the workplace, cronies behave differently than non-cronies toward their supervisors. For instance, the cronies are more motivated to repay favors with favorable attitudes and behaviors such as organizational commitment, organizational citizenship behavior, and ingratiation. In contrast, non-cronies are discriminated against and they respond with negative behaviors and attitudes such as deviant workplace behavior, low organizational commitment and turnover intentions, and job dissatisfaction (Nadeem et al., 2015). Despite much evidence in the current literature that organizational cronyism results in severe individual and organizational consequences, there are few studies on attitudinal reactions of organizational cronyism and how the notion of organizational cronyism translates into attitudinal outcomes (Turhan, 2014).

The relational psychological contract is a dimension of psychological contract (Bari et al., 2016). Rousseau (1989) defines a psychological contract as “an individual’s beliefs about the terms of the exchange agreement between employee and employer.” Rousseau also categorizes psychological contract into relational psychological contract, transactional psychological contract, and a balanced psychological contract (Hui et al., 2004). The relational psychological contract refers to long-term employment relations with a wider scope, reciprocal trust, and loyalty, containing both monetizable and socio-emotional features such as mentoring support (Hui et al., 2004; Suazo, 2009). Mostly in relational psychological contracts, seniority decides career growth and financial benefits as compared to work performance. Employees love long-term employment relationships and direct participation in organizational development. Employees are physically and emotionally committed to the organization after relational psychological contract development. The relational psychological contract between leaders and employees develops ingratiation in cronies.

The study provides a significant addition to the existing body of knowledge in several ways. First, the current study has been conducted in public sector organizations of Pakistan, where much attention has been given to relationships instead of employee’s actual knowledge skills and expertise (Bashir and Nasir, 2013). The study shed a light on unexplored reasons for employee ingratiation at the workplace, which is known as organizational cronyism. The concept of organizational cronyism is very popular in the public sector, but there are few empirical pieces of evidence regarding this most observable fact (Turhan, 2014; Saleem et al., 2018). Second, the study attempts to explain the impact of organizational cronyism on employee ingratiation through relational psychological contract. By doing so we tried to establish a complete path which explains that employees who get favor from their leaders/supervisors are much more enthusiastic to build long term relationships with the favor-giver so that they could enjoy long-term benefits. Employee ingratiation behavior is much accepted and valued in the public sector of Pakistan (Bashir and Nasir, 2013; Shaheen et al., 2017). For this reason, cronies enjoy exceptional favor and support from their leader. There is an abundant literature on employee ingratiation, but the reason for organizational cronyism is missing in current literature (Khatri and Tsang, 2003). Moreover, the study is also an attempt to answer the call of Turhan (2014) who proposed the empirical investigation of the reasons and causes of organizational cronyism at the workplace due to its recurrent practice in the public as well as private sector. Drawn on social exchange theory (Blau, 1964; Adams, 1965), the employees who receive favor, respect, and trust from their leaders or supervisors are more motivated to establish long-term relations with their leaders as well as with their organizations. The trust and mutual understanding between cronies and leaders provide a base for relational psychological contract formation and development.

The concern of this study is to address this gap by investigating the relationship between organizational cronyism and employee attitudinal outcome, i.e., ingratiation. Along with testing the main relationship between organizational cronyism and ingratiation, the present study also aims to clarify the mechanism between organizational cronyism and ingratiation by answering the question of how the notion of organizational cronyism translates into employee ingratiation.

Hence, the objective of the study is to investigate the impact of organizational cronyism on employee ingratiation. Existing studies are on behavioral outcomes of organizational cronyism and, to the best of the authors’ knowledge, this is the first study to examine the attitudinal outcomes of organizational cronyism in public sector organization of Pakistan. Second, by explaining the relationship between organizational cronyism and employees’ ingratiation, the present study offers a detailed view of how organizational cronyism translates into attitudinal outcomes. Third, the high-power distance culture is common in public sector organizations of Pakistan, and the cronies choose ingratiatory tactics to receive support from senior management. Therefore, the present study is conducted in public sector organizations of Pakistan such as defense production, human rights, parliamentary affairs, petroleum, and natural resources.

To seek the above-mentioned objectives, the present study has the following research questions. What is the effect of organizational cronyism on employees’ ingratiation? And how does the relational psychological contract mediate the relationship between organizational cronyism and employees’ ingratiation?

## Theoretical Framework and Hypotheses Development

### Cronyism and Ingratiation

The word cronyism is derived initially from Greek word “khrónios” then it changed into “crony” in English. The meanings of “khrónios” are long-standing, enduring and long term (Turhan, 2014). In the Oxford dictionary, the word “crony” is described as a friend, a friend of long standing or companion (Oxford Dictionary and Thesaurus, 1999). The term cronyism was coined first time in 1984, which means “the extreme passion and skills to make friends” (Khatri et al., 2006). However, in 1952 in the United States, the term cronyism was used in a political sense when the Truman administration was alleged to select employees within the official postal administration based on close relations rather than objective criteria. Afterward, cronyism started to be considered as companionship or synchronization-based type of favoritism, and this conception about cronyism changed the purity of the word altogether (Khatri and Tsang, 2003).

The term organizational cronyism is a popular term in political science literature. However, Bankston (2014) explained that social and business organizations are also political arenas and general organizations cannot be considered free from such practices. Therefore, several investigators link cronyism with business and social organizations as well. Organizational cronyism is defined as exploiting power and resources for bestowing privileges onto relatives and friends. In organizational cronyism merit violation is a common practice and the decisions are made on the basis of subjective grounds rather than objective ones (Rynes et al., 2005; Arasli and Tumer, 2008). In the presence of organizational cronyism, certain employees enjoy a comfortable working environment, high ratings in selections, promotions, and appraisal procedures as well as challenging assignments. In return, they are more persuaded to respond with positive behaviors and attitudes, such as organizational commitment and job satisfaction (Chen et al., 2013; Cai et al., 2018). In addition, the employees who are treated as cronies try to establish a harmonious relationship with the favor-giver by displaying conformity and a “Yes, sir” attitude towards the supervisor or leaders, and such types of tactics are called ingratiation (Schriesheim and Hinkin, 1990). Ingratiation refers to an intentional attempt to control and influence others or becoming amiable in the eyes of the target (Keeves et al., 2014; Liu et al., 2014; Luo et al., 2014; Sibunruang et al., 2016).

The present study is interested in investigating the relationship between organizational cronyism and ingratiation. In organizational cronyism culture, subjective decision-making behavior of a supervisor ultimately boosts employee ingratiation at the workplace. Drawn on social exchange theory (Blau, 1964), the employees who receive favor, support, and trust from their supervisors, as a result, are more willing to repay the favor with likable attitudes such as ingratiation, and sometimes break the organizational system only for the happiness of favor giver. Hence, this study hypothesized that;

**H1:** Organizational cronyism has positive effects on ingratiation

### Organizational Cronyism and Relational Psychological Contract

The term psychological contract has been known as mutual commitments and obligations between employee and employer. It is also known as an exchange relationship between two parties (e.g., employee and employer). The psychological contract was been introduced in the 1960s by Argyris (1960), Levinson et al. (1962), Schein and Bennis (1965). After that, the psychological contract has been continuously maintaining its attraction in the eyes of researchers, and a wide range of antecedents and outcomes of the psychological contract have been investigated. Researchers have also identified its dimensions, such as transactional psychological contract, relational psychological contract, a balanced psychological contract, and a transitional psychological contract (Rousseau, 1989, 1995; Robinson and Morrison, 2000). However, the first two dimensions i.e., transactional and relational psychological contracts, have gained more attention from scholars (Raja et al., 2004: Yu and Chen, 2008).

The present study considered the relational psychological contract as a mediator on the following grounds. The relational psychological contract differs from the transactional psychological contract in terms of timeframe (e.g., long term vs. short term), the durability of relationships, reciprocation, and expectations. One key reason for relational psychological contract selection is “friendship.” A transactional psychological contract does not cover the element of friendship. It has minimum future assistance without close contacts between the parties and is based on monetary terms and short-term relations (Conway and Briner, 2005; Isaksson et al., 2010; Yang et al., 2016).

In contrast, the relational psychological contract is based on long term relations, open-ended relations, informal discussions and open communication (Millward and Brewerton, 2000). The relational psychological contract is treated as a contract beyond written formalities and consists of interpersonal relationships (Morrison and Robinson, 1997). Moreover, the relational psychological contract consists of friendships and associations which lead to positive workplace behaviors. Hence, the relational psychological contract is purely based on strong associations and demands long-term commitments (Chaudhry and Tekleab, 2013; Guchait et al., 2015). On the other hand, organizational cronyism is purely based on informal relationships, associations and informal commitment, which demands exceptional bonding (Saleem et al., 2018). Hence, both (favor-giver and favor-taker) expect a two-way relationship, thus expectations are required on both sides—for the employee as well employer (Khatri et al., 2006). When the employer/leader provides undue favor to an employee as a result, the employer also expects an association, praise, and commitment from the employee (Chen et al., 2013; Turhan, 2014). In organizational cronyism, cronies enjoy relaxation in assignments, flexible working hours, trust and support from the employer (Arasli and Tumer, 2008). To enjoy these benefits for a longer period of time, cronies attempt to establish a long-term relationship with the favor-giver, and thus relational psychological contract emerges. Therefore, we use the notion of relational psychological contract to explain the link between organizational cronyism and ingratiation.

Khatri et al. (2006) explained cronyism through the lens of social exchange theory. Khatri et al. (2006) argued that cronyism is a reciprocal exchange transaction where party A favors party B on the basis of the relationship that exists between them in a social network at the expense of party C’s equivalent or larger claim to the valued resource. In this scenario, one group of employees receive undue favor, support and reward by the leader based on their mutual relations, while another group is discriminated against (Mucci et al., 2015). The employees who are discriminated and do not get rewards, they perceive leaders and organization are dishonest. The employees’ feelings of prejudices incite and lead them toward a relational psychological contract breach (Robinson and Morrison, 2000; Uhl-Bien and Maslyn, 2003; Giorgi et al., 2013; Ertas, 2015). On the other hand, employees who gain trust, support and rewards from the supervisors, repay the organization by displaying long-term relationships and loyalty toward organization and supervisor (Qi et al., 2019). Therefore, the present study considers that organizational cronyism promotes relational psychological contract.

In the presence of organizational cronyism, the elements of friendship, long-term relationships, and connections are utmost. Similarly, friendship and long-term relationship are also fundamental elements of the relational psychological contract (Chaudhry and Tekleab, 2013). Social exchange theory (Blau, 1964) also provides a strong theoretical basis to establish a positive link between organizational cronyism and the relational psychological contract. According to social exchange theory, positive acts are recompensed by positive behaviors and vice versa. Hence, the present study hypothesis:

**H2:** Organizational cronyism has a positive association with relational psychological contract

### Relational Psychological Contract and Ingratiation

The employees having relational psychological contract attempt to strengthen the relationship with the organization and leaders by adopting positive behaviors such as long-term commitments and extra-role behaviors (Kwon Choi et al., 2014). The relational psychological contract transpires when employees encounter a positive exchange of transactions with the employer (Jepsen and Rodwell, 2012). When an employer provides above average benefits, trust, and support, the employees adopt ingratiation tactics and try to enhance their likelihood in the eyes of the employer. Through ingratiation tactics, subordinates establish a long-term relationship with the leader. Ingratiation is defined as “an intentional attempt to increase one’s likelihood in the eyes of others” (Liden and Mitchell, 1988). An empirical study conducted by Tripathi (1990) in public sector organizations of India found that good quality of relationships between employee and employer depends upon ingratiation.

In social exchange, when employees receive preferential treatment from employers, in response, they repay through their ingratiatory behavior to establish an enduring relationship with the employer. Using social exchange theory as a theoretical basis, this study considers that there is a positive relationship between relational psychological contract and ingratiation. Therefore, it is proposed that;

**H3:** Relational psychological contract has positive effects on ingratiation

### The Mediating Role of Relational Psychological Contract

According to Cropanzano and Mitchell (2005), social exchange theory has been used as a grounded supporting theory in explaining workplace relationships. Several scholars have explained that psychological contract is based on the social exchange theory, i.e., rewards, status, and recognition to employees is a response to their contribution in the organization performance (Cropanzano and Mitchell, 2005; Liden and Mitchell, 1988). As per the norms of positive reciprocity, if someone is doing a favor to another and fulfilling his/her expectations accordingly, reciprocally, positive behaviors and attitudes are performed in response to this favor (Peyton et al., 2019; Su et al., 2019). For instance, if the supervisor appreciates the subordinate’s actions and provides them better working opportunities with support, in response, subordinates reciprocate it with long-term commitments, respect and positive words (Vincent-Höper and Stein, 2019; Zhang et al., 2019).

Scholars argue there are a number of antecedents of the relational psychological contract (Cavanaugh and Noe, 1999; Robinson and Morrison, 2000). Employees are motivated to build relational psychological contract for a number of reasons, such as organizational support, leader’s trust, and support and challenging working environment. The leader’s trust and favor motivate the employee to establish long-lasting relationships with the organization. According to leader-member exchange theory, employees who are favored by leaders prefer to maintain long-term relationships with the leader as well as with the organization (Turnley et al., 2003; Martin et al., 2015).

In the presence of organizational cronyism, the distinction between ingroup and outgroup is also noticeable. The leader provides challenging assignments, flexible working hours, trust and support to those who belong to him or her in any way, while others are discriminated against. Hence, favored and discriminated employees have different attitudes and behaviors toward their organization (Schriesheim et al., 1999). Those who are discriminated against or non-cronies encounter psychological contract breach while favored employees wish to maintain positive and long-term relationships with the leader as well as with the organization in response to their favor and benevolence. Consequently, in a struggle to maintain a relational psychological contract with the favor-giver, employees choose to impress them by demonstrating influential tactics such as ingratiation (Turhan, 2014). Therefore, we propose a relational psychological contract as an explanatory mechanism in the relationship between organizational cronyism and ingratiation.

**H4:** Relational Psychological Contract Mediates the Relationship Between Organizational Cronyism and Ingratiation

**FIGURE 1:**
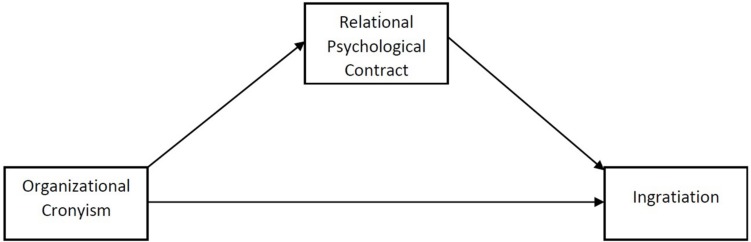
Study framework.

Figure 1 explains the study framework and the relationship between the variables.

## Materials and Methods

### Sample and Data Collection

The population of this study is the employees working in different ministerial offices such as the ministry of defense production, human rights, parliamentary affairs, petroleum, and natural resources in Islamabad, Pakistan. In order to recruit participants and to control their social desirability bias (i.e., the tendency of survey respondents to answer the questions in a manner that will be viewed favorably by others), the following procedure was pursued. The authors visited the work sites and briefly explained the subject of the study. The authors contacted the principal officers of human resource departments and explained to them the purpose of the data collection. In exchange, the authors promised to share the findings and managerial implications of the study upon request.

The data collection process was conducted in two waves, 8 weeks apart. The two surveys collected at respectively, Time 1 and Time 2 contained unique identification numbers because authors could match the responses collected in each wave. Before data collection, the principal author visited the public sector organizations and received permission from the heads of the ministries and requested them to motivate their employees to provide their valuable response in each wave. Moreover, the current study was also approved by the ethics committee of the institute from which the authors belong (Lyallpur Business School, Faisalabad, Government College University, Faisalabad, Pakistan).

A cover letter that explained the purpose of the study and gave assurance that the data of all respondents would be kept confidential and would be accessible only by the authors. The information at the individual level would never be made public, and only aggregate data would be used in the research. Moreover, the cover letter also assured the respondents that there are no correct or incorrect answers and stated that the respondents should answer the questions as naturally as possible. These measures helped to reduce social desirability or acquiescence biases (Spector, 2006). Additionally, data were collected on the consent and willingness of the respondents without any social or professional pressure. The first survey assessed employees’ perceived climate of cronyism. The second survey then asked these same respondents to fill questionnaires regarding ingratiation and the relational psychological contract. The informed consent of the contributors was implied through survey accomplishment.

From 400 surveys, we received back 310 complete pairs of surveys across the two data collection points, the response rate is 77.5%. In organizational sciences literature, a few pieces of evidence are available regarding response rate. Baruch and Holtom (2008) recommended that a 60% response rate is an acceptable figure. Thus, the response rate of the present study is acceptable and allows the authors to proceed for data analysis (see Baruch and Holtom, 2008). The present study also addressed the question of non-response bias potential. The authors solved this concern by comparing early to late respondents (Dillman, 1991). By comparing the results of the group of early respondents to the group that only responded after being sent a follow-up reminder, the authors found no significant differences in responses. These results add additional support to the quality of the data and subsequent findings of the present study.

Because employees of public sector organizations are good in the English language and their medium of exchange in their offices is the English language, the questionnaires were therefore distributed and measured in English. This approach aligns with other studies conducting research in Pakistan (e.g., Raja and Johns, 2010; Abbas et al., 2014; Butt et al., 2017).

### Demographics

Among a total of 250 employees, 1.2% are intermediate, 32% are bachelors, and 66% are masters. Likewise, 2.4% of employees have less than 1 year of experience, 4.6% have 1–2 years of experience, 3.6% have 2–3 years of experience, and 92.4% have above 3 years of experience. In the case of gender, 76.8% are male and 32.2% are females. Moreover, 44% of employees fall in 20–30 years of age category, 40% are 30–40, 13.2% are above 40 and 2.8% have aged more than 50 years.

### Measures

We used previously validated items to measure the focal constructs. All items are measured at five-point Likert scales ranging from 1 (strongly disagree) to 5 (strongly agree), unless otherwise indicated.

#### Organizational Cronyism

Organizational cronyism is measured by adopting a 15-item scale developed by Turhan (2014). Sample items of organizational cronyism are “Our manager treats employees with whom he has a closer personal connection with more tolerance” or “When resolving conflicts, our manager protects employees with whom he has a closer personal connection.” Cronbach’s alpha reliability for organizational cronyism is adequate (Cronbach’s α = 0.84).

#### Relational Psychological Contract

The relational psychological contract was measured by using a scale developed by Millward and Hopkins (1998). The fifteen-item scale of relational psychological contract was adapted to measure the employees’ long-term relationships as well as expectations of employees from the organization. The sample scale items are “This job is a stepping stone in my career development,” “I expect to develop my skills (via training) in this company,” and “I expect to gain promotion in this company with the length of service and effort to achieve goals.” Cronbach’s alpha reliability for relational psychological contract was adequate (Cronbach’s α = 0.73).

#### Ingratiation

Employees’ ingratiatory behaviors were measured by using an 11-item scale developed by Kipnis et al. (1980). The sample scale items are “Made him or her feel important (“only you have the brains, talent to do this”),” “Acted very humbly to him or her while making my request,” and “Acted in a friendly manner prior to asking for what I wanted.” Cronbach’s alpha reliability for relational psychological contract was adequate (Cronbach’s α = 0.82).

#### Statistical Model

AMOS is known as analysis of a moment structure and it is particularly used for structural equation modeling SEM.

We used AMOS to perform statistical analysis due to various reasons. First, the statistical analysis could be executed excellently, precisely and proficiently. Second, AMOS is a co-variance-based SEM (Hair et al., 1998). SEM is used for theory testing; in the current study we test theory, therefore we chose to apply AMOS on our proposed framework. Secondly, SEM is based on two stages—first, measurement model, and second, structural model. In the measurement model, confirmatory factor analysis was performed to check the association of a latent variable with its items prior to the investigation of the structural model (Hair et al., 2006). In the structural model, the impact of direct and indirect relationships among latent variables was inspected. Therefore, we believe AMOS to be a perfect statistical tool to inspect our proposed model.

## Results

### Control Variables

In order to check significance difference across outcome variable one-way ANOVA was performed on the collected data. As per the results, the authors found insignificance difference across qualification (*F* = 0.895; *P* > 0.05), experience (*F* = 0.584; *P* > 0.05), gender (*F* = 1.48; *P* > 0.05) and age (*F* = 1.20; *P* > 0.05). Hence, there is not any control variable in our study.

### Correlation Analyses

Descriptive statistics of all theoretical variables and their correlations have been reported in Table 1. As per the results, organizational cronyism is positively and significantly related with the relational psychological contract (*r* = 0.0174, *p* < 0.05) and ingratiation (*r* = 0.589, *p* < 0.05), and the relational psychological contract is positively related with ingratiation (*r* = 0.306, *p* < 0.05).

**TABLE 1:** Correlation analysis.

**Variables**	**Mean**	**SD**	**1**	**2**	**3**	**4**	**5**	**6**	**7**
Qualification	3.65	0.50	1						
Tenure	3.86	0.54	0.088	1					
Gender	1.23	0.42	0.132^*^	0.19	1				
Age	1.74	0.78	−0.037	0.95	−0.257^∗∗∗^	1			
CRN	3.51	0.58	0.057	−0.038	−0.019	0.049	(0.84)		
RC	2.69	0.45	0.096	−0.018	−0.12	−0.066	0.174^*^	(0.73)	
ING	3.51	0.75	0.092	−0.119	−0.099	−0.012	0.589^*^	0.306^∗∗^	(0.82)

^*^ = *P* < 0.001; ^∗∗∗^*P* < 0.05; CRN, organizational Cronyism; RC, Relational Contract; ING, Ingratiation.

### Confirmatory Factor Analysis

In order to test the measurement model, confirmatory factor analysis was performed before testing the hypothesis through AMOS 23. Model fitness is assessed through IFI, TLI, comparative fit index (CFI) and root mean square error of approximation (RMSEA). The proposed model has three theoretical variables: one independent variable, one mediating variable, and one dependent variable. It is evident from Table 2 that initial model fitness is very poor because all values for IFI, TLI, comparative fit index (CFI) and root mean square error of approximation (RMSEA) do not meet threshold boundaries. Therefore, a series of modifications have been performed to achieve a good model of fitness. After a number of modifications, we achieved good model fitness reflected by values of IFI = 0.981, TLI = 0.903, CFI = 0.917 and RMSEA = 0.061.

**TABLE 2:** Confirmatory factor analysis.

	**Chi Square**	**df**	**CMIN/DF**	**IFI**	**TLI**	**CFI**	**RMSEA**
Initial Model	2527.514	725	3.486	0.441	0.404	0.438	0.152
Modified Model	316.203	151	2.094	0.981	0.903	0.917	0.061

### Hypotheses Verification

Table 3 demonstrates the relationship between independent variable organizational cronyism and dependent variable ingratiation (β = 0.553, *p* < 0.001). Hence, H1 is supported. Moreover, organizational cronyism is positively and significantly related to relational psychological contract, which is proved with regression coefficients (β = 0.174, *p* < 0.05); thus, H2 is accepted. H3 was examined by investigating the impact of the relational psychological contract on ingratiation (β = 0.210, *p* < 0.05). Consequently, H3 is also supported.

**TABLE 3:** Structural path coefficients.

**Structural Paths**	**Path Coefficients**	**SE**	***P*-value**
Cronyism → Ingratiation	0.553	0.065	^∗∗∗^
Cronyism → Relational Contract	0.174	0.048	0.005
Relational → Contract Ingratiation	0.210	0.083	^∗∗∗^

### Mediation Analysis

**FIGURE 2:**
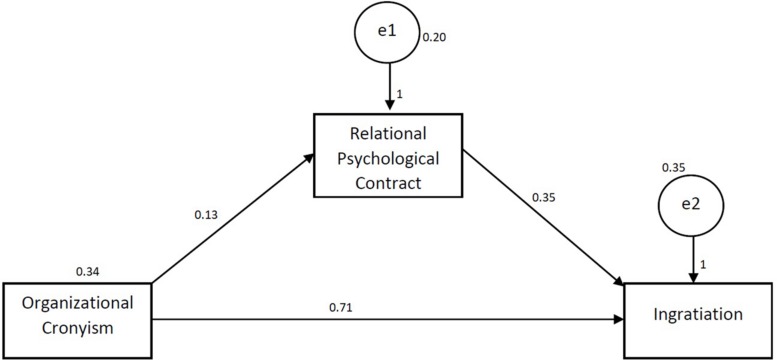
Post-Analysis Study Model.

The obtained results of hypothesis 4 confirmed a mediating role of the relational psychological contract in the relationship between organizational cronyism and ingratiation, reflected by the direct and indirect effect in Table 4. The relationship of organizational cronyism in the presence of a relational psychological contract is still significant (β = 0.036, *p* < 0.05) but the relationship has been reduced in presence, hence proving partial mediation. Therefore, relational psychological contract partially mediates the relationship between organizational cronyism and ingratiation. Figure 2 explains the post-analysis study model.

**TABLE 4:** Mediation analysis.

**Hypothesis**	**Direct effect**	**Indirect effect**	**Result**
CR → RPC → ING	0.553^∗∗∗^	0.036^*^	Partial Mediation

^*^ = P < *0.05; ^∗∗∗^P* < *0.001; Bootstrap sample size 5000; CR, Cronyism; RC, Relational psychological Contract; ING, Ingratiation.*

## Discussion

Recently, organizational cronyism gained much attention from academic researchers and professionals due to its adverse consequences. By contributing to the existing body of knowledge, the authors investigate organizational cronyism as an antecedent of ingratiation. The present study also examined the relational psychological contract as a mediating mechanism in the relationship between organizational cronyism and ingratiation. The present study found good support for our hypothesis. According to the results of this study, organizational cronyism is significantly related to relational psychological contract and ingratiation, and relational psychological contract partially mediates the relationship between organizational cronyism and employees’ ingratiation. The findings of the current study are in line with previous studies which suggest that employees who receive undue favor, trust, support and reward from their supervisors are more motivated to display influential tactic such as ingratiation (Deluga and Perry, 1994; Kim and George, 2005; Dulac et al., 2008; Turhan, 2014; Kauppila, 2016).

Organizational cronyism is prevalent in the culture of every organization, though some cultural norms and values provide an encouraging environment to flourish such practices at the workplace, such as high-power distance cultures (Khatri et al., 2006; Turhan, 2014; Al-Shawawreh, 2016). In public sector organizations of Pakistan, a high power distance exists; therefore, employees try to resolve their issues by adopting “yes, sir” attitude. Moreover, the supervisor also trusts and supports those employees who show confirmation and compliance with their decision and acknowledge their superiority. Therefore, it is the common perception of employees in public sector organizations of Pakistan that employees who demonstrate ingratiatory attitude to influence supervisor’s decision will get privileges during allocation of rewards and performance appraisal (Bashir and Nasir, 2013).

The construct of organizational cronyism is very common in public sector organizations of Pakistan, and employees are in a struggle to become part of the ingroup of the supervisor so that they can get benefits from their supervisors. As a result, influential tactics have been practiced and employees seem to be in a race to build a long-lasting and harmonious relationship with their supervisor in any way. Pakistani public sector organizations are characterized as high in corruption and lack merit-based decisions due to a weak culpability framework (Bashir and Nasir, 2013; Shaheen et al., 2017). Such organizations demand attention from academic researchers, but existing studies are restricted to only multinational companies and western cultures. The current study not only presented a true picture of organizational cronyism in public sector organizations of Pakistan but also provides complete empirical evidence of how practices of organizational cronyism result in employees’ influencing tactics such as ingratiation.

The current study depicts a true picture of public sector organizations of Pakistan. The conclusion of the study helps us in understanding that there exists a motivation behind employee ingratiatory behavior, and one fundamental source of success in the public sector is the support of and relationships with the leader. Cronies’ better excel and shine at the workplace as a contrast to non-cronies due to having relationships with the leader as well as by effectively applying ingratiatory tactics. Second, employees who are recruited and selected based on non-performance related factors use unprofessional ways to survive at the workplace, and instead of lack of skills, knowledge, and expertise they enjoy above average benefits such as relaxation in assignments, flexible working hours, health and breaks amenities. We found organizational cronyism is a fundamental reason for giving birth to these unhealthy workplace behaviors. Moreover, the current study also fills the gap in the literature by answering the call of Turhan (2014) and Khatri (2016), who call to investigate employee ingratiation as a result of organizational cronyism. The study also paints a clear picture of public sector institutions of Pakistan, where relationships are given more importance as a contrast to actual knowledge, skills and expertise, and employees are in a struggle to maintain harmonious relationships with the leader instead of concentrating on their tasks.

### Theoretical and Managerial Implications

The current study has some theoretical implications. The authors tried to enrich the organizational cronyism literature. For this purpose, the authors tested the impact of organizational cronyism on employees’ ingratiatory behaviors through the lens of the relational psychological contract. The empirical investigation of organizational cronyism and ingratiation also addressed a gap in the existing literature which was highlighted by Khatri (2013). Moreover, these indispensable links have been supported with the help of social exchange theory. Previous studies tested only the negative impact of organizational cronyism such as job dissatisfaction, deviant workplace behavior and breach of psychological contract (Aydogan, 2009; Turhan, 2014; Fu, 2015; Jones and Stout, 2015; Al-Shawawreh, 2016). The current study states that cronies develop a relational psychological contract with favor-givers—in return, the employees are more motivated to uphold compliance with their leader, which fulfills both the leader’s and subordinate’s interests. As a result, a crony demonstrates more positive behavior and less negative behavior.

The results of the current study have several implications for the organizations which have been discussed below. According to the results of the study, organizational cronyism encourages employees to indulge in ingratiatory tactics as an effort to receive favor and support as well as to maintain pleasant relationships with a supervisor in the future. By doing so, cronies get privileges over non-cronies which can damage the well-being of other employees. One of the most suitable ways to stop such practices is to allow balanced and merit-based decision making to flourish in organizations. The managers, especially in public sector organizations, must understand that today’s workforce in the public sector is very aware of their rights and are not ready to face inequity in any case; hence they should prepare themselves to face the brutal consequences of organizational cronyism if they do not stop such practices. Another suitable way is to motivate top management in flourishing merit-based decisions in public organizations rather than an influential one. There should be a proper platform for employees to make them sure and understand that in organizations only merit-based decisions will be made. This could be done only if top management demonstrates fair decision making in hiring, selection, and reward allocation procedures.

### Study Limitations, Strengths, and Future Research Directions

There are also certain limitations to this study. The present study investigated only one consequence of organizational cronyism, while a number of other related important outcomes require the attention of researchers. In the future, researchers may investigate the attitudinal and behavioral outcomes of organizational cronyism such as organizational commitment, organizational citizenship behavior, and job satisfaction. Second, the present study chose a limited sample size, and the findings of the study could be more robust and more generalizable with a large sample size. Moreover, there are also a number of other factors which may help in understanding how the notion of organizational cronyism translates into attitudinal outcomes such as leader-member exchange and culture of an organization and state. The current study only addresses the individual outcome of organizational cronyism, more diverse findings could be reported if the impact of organizational cronyism is examined at the organizational level. Further, the role of organizational cronyism on organizational performance could be tested with both positive and negative parameters, for example the impact of organizational cronyism on organizational performance can be examined through both ingroup and outgroup employees with various moderators such as different cultural variations and personality types.

Despite having the above-mentioned limitations, the current study has a number of strengths. First, the current study is a significant addition to the existing body of OC literature. The practices of OC are very common and observable everywhere in Pakistan, but there are a few empirical pieces of evidence in the literature. Second, the authors present a clear picture of how employees in the public sector of Pakistan took benefit from informal networks and connections. Third, the study has been conducted in public sector organizations of Pakistan, and public sectors are usually victims of such dishonest practices. Forth, the study also fulfills the gap in the current literature highlighted by Khatri (2013) and Turhan (2014).

## Conclusion

The present study helps us in understanding that there exists a motivation behind employee ingratiatory behavior and the one fundamental source of success in the public sector is the support of and relationships with the leader. Cronies better excel and shine at the workplace as a contrast to non-cronies due to having a mature relationship with the leader as well as by effectively applying ingratiatory tactics. Second, employees who are recruited and selected based on non-performance related factors use unprofessional ways to survive at the workplace. Even if they have a lack of skills, knowledge, and expertise they enjoy above average benefits such as relaxation in assignments, flexible working hours, and health and breaks amenities. We found organizational cronyism is a fundamental reason for giving birth to these unhealthy workplace behaviors. The study also paints a clear picture of public sector institutions of Pakistan where relationships are given more importance in contrast to actual knowledge, skills and expertise, and employees are in a struggle to maintain harmonious relationships with the leader instead of paying concentration on their tasks.

## Data Availability

The datasets for this manuscript are not publicly available because authors have a commitment with participants to not share their answers/data. Requests to access the datasets should be directed to muhammadwaseembari786@hotmail.com.

## Author Contributions

SS developed the idea and research design, drafted the manuscript. MWB helped in data collection and performed the analysis. FH and MMA helped in revision of the manuscript such as restructuring of the paper, improving the theoretical framework of the manuscript, final revision, and editing of the paper. All authors jointly revised and edited several times final version of the manuscript.

## Conflict of Interest Statement

The authors declare that the research was conducted in the absence of any commercial or financial relationships that could be construed as a potential conflict of interest.
